# COVID-19-associated encephalopathy: connection between neuroinflammation and microbiota-gut-brain axis

**DOI:** 10.3389/fmicb.2024.1406874

**Published:** 2024-05-28

**Authors:** Khrystyna Duve, Pavlo Petakh, Oleksandr Kamyshnyi

**Affiliations:** ^1^Department of Neurology, I. Horbachevsky Ternopil National Medical University, Ternopil, Ukraine; ^2^Department of Biochemistry and Pharmacology, Uzhhorod National University, Uzhhorod, Ukraine; ^3^Department of Microbiology, Virology, and Immunology, I. Horbachevsky Ternopil National Medical University, Ternopil, Ukraine

**Keywords:** encephalopathy, gut microbiome, COVID-19, gut-brain axis, probiotic, cytokine, inflammation, butyrate

## Abstract

While neurological complications of COVID-19, such as encephalopathy, are relatively rare, their potential significant impact on long-term morbidity is substantial, especially given the large number of infected patients. Two proposed hypotheses for the pathogenesis of this condition are hypoxia and the uncontrolled release of proinflammatory cytokines. The gut microbiota plays an important role in regulating immune homeostasis and overall gut health, including its effects on brain health through various pathways collectively termed the gut–brain axis. Recent studies have shown that COVID-19 patients exhibit gut dysbiosis, but how this dysbiosis can affect inflammation in the central nervous system (CNS) remains unclear. In this context, we discuss how dysbiosis could contribute to neuroinflammation and provide recent data on the features of neuroinflammation in COVID-19 patients.

## 1 Introduction

COVID-19, caused by the SARS-CoV-2 virus, is linked to various neurological complications, including COVID-19 encephalopathy, which is often observed in severe cases, particularly in older patients with acute respiratory distress syndrome and multiple organ failure (Ellul et al., 2020; Abenza Abildúa et al., 2021; Garg et al., 2021; Pilotto et al., 2021; Akimoto et al., 2022). The Global COVID-19 Neuro Research Coalition defines COVID-19 encephalopathy as necessitating a COVID-19 diagnosis, temporal correlation with symptoms, exclusion of other diseases, and differentiation between primary brain disease and secondary brain dysfunction related to COVID-19 severity (Michael et al., 2023).

Studies on the human microbiome have revealed its crucial role in immune system development, combating pathogens, toxin elimination, metabolic functions, and overall health (Gu et al., 2020; Villapol, 2020). Research indicates that COVID-19 patients exhibit reduced gut microbiome diversity and abundance, with an increase in opportunistic pathogens and a decrease in beneficial microbiota, correlating with respiratory issues and neuropsychiatric symptoms (Wu et al., 2021). The complex connection between the gut and brain, known as the microbiota-gut-brain axis (MGBA), involves interactions through intestinal cells, the enteric nervous system (ENS), metabolic pathways, and neuroendocrine mechanisms, as supported by numerous clinical studies (Cryan et al., 2019; Morais et al., 2021).

The neurological manifestations linked to COVID-19 are driven by inflammatory responses that inundate the brain via proinflammatory factors, causing damage to neural cells and resulting in brain ischemia (Otani et al., 2023). The gut microbiota plays an important role in controlling inflammation through direct and indirect mechanisms (Maciel-Fiuza et al., 2023). However, there is very little information about the role of neuroinflammation in COVID-19 encephalopathy and the potential role of the gut microbiota in regulating inflammation. With this review, we aim to elucidate this gap to provide possible new therapeutic options and understand the pathophysiology more comprehensively.

## 2 Neuroinflammation in COVID-19 encephalopathy

### 2.1 Symptoms and pathogenesis

More than half of hospitalized patients exhibit symptoms such as dizziness, altered mental status, ataxia, and cognitive dysfunction, with encephalopathy recognized as the most prevalent neurological symptom of COVID-19 (Graham et al., 2022). Currently, two main hypotheses regarding the pathogenesis of COVID-19 encephalopathy have been established: the hypoxic hypothesis and the inflammatory cytokine-based hypothesis (Ahmad and Rathore, 2020).

### 2.2 Hypoxic hypothesis

Systemic hypoxia resulting from COVID-19 infection can lead to metabolic deficiencies, causing global brain dysfunction. COVID-19 disrupts respiratory function, impairing gas exchange in the lungs and potentially inducing a hypoxic state (Vengalil et al., 2023). The metabolic processes of brain tissue are dependent on oxygen, and insufficient oxygen levels impede the oxidation of glucose for the production of ATP. An insufficient amount of energy can result in impaired neuronal function and ultimately cellular death, thereby playing a role in the development of COVID-19 encephalopathy (Karuppan et al., 2021).

The interaction between the brain and lungs, known as the brain-lung crosstalk axis, is a crucial but often overlooked mechanism that has significant implications for ventilatory management in the development of brain complications associated with COVID-19. Decreased systemic oxygen levels can impact the oxygenation of brain tissue, potentially leading to secondary brain injury. Concurrently, disturbances in lung function can disrupt the delicate equilibrium between oxygen and carbon dioxide levels, which are pivotal for maintaining cerebral homeostasis (Robba et al., 2021). Such disruptions can result in alterations in cerebral blood flow, leading to either ischemic or hyperemic conditions within the brain (Battaglini et al., 2020). These vascular changes may eventually culminate in cerebral edema and impairment of cerebral autoregulation. Notably, postmortem examinations of COVID-19 patients have consistently revealed acute hypoxic damage to both the cerebrum and cerebellum in the absence of evidence suggesting direct brain invasion or encephalitis (Solomon et al., 2020).

### 2.3 The cytokine-based hypothesis

The second mechanism involves the uncontrolled proliferation and secretion of cytokines triggered by COVID-19 infection (Perrin et al., 2021). This idea was supported by the findings of a clinical trial by Dirk Reinhold et al., who reported that COVID-19 is not primarily a neuroinflammatory disease; however, it does impact the brain indirectly by activating inflammatory pathways outside of the brain (Reinhold et al., 2023). The release of cytokines can trigger a series of reactions, which can weaken the integrity of the endothelial lining and disrupt the blood–brain barrier (BBB) (Perrin et al., 2021). In the blood of COVID-19 patients, researchers have found significantly increased levels of cytokines such as interleukin-6 (IL-6) and the astroglial marker S100B. Elevated levels of S100B are associated with increased permeability of the BBB, while elevated levels of IL-6 suggest CRS in the brain. After crossing the BBB, IL-6 triggers a positive feedback mechanism, stimulating adjacent immune cells to release additional cytokines such as IL-1β, TNF-α, and IFN-γ (Vengalil et al., 2023). These proinflammatory cytokines further activate brain-resident macrophages, perpetuating a secondary inflammatory response in brain tissue. This ongoing inflammation triggers the release of more cytokines, intensifying the positive feedback loop. Thus, monocytes and macrophages cross the BBB and initiate reactive gliosis, a process characterized by the proliferation of reactive glial cells, leading to scarring and the onset of encephalopathy.

A study conducted in France (Strasbourg) focused on COVID-19 patients who exhibited severe neurological symptoms, including confusion, cerebellar ataxia, agitation, tremor, and impaired consciousness (Vengalil et al., 2023). Laboratory evaluations were performed to assess CRS markers, such as IL-6, C-reactive protein, ferritin, and lactate dehydrogenase. In more than 50% of the patients, the highest levels of these markers occurred simultaneously with the emergence of neurological symptoms. Furthermore, an increase in the permeability of the BBB, as evidenced by elevated levels of S100B protein during CRS, was observed. Notably, reverse transcription PCR assays were unable to detect SARS-CoV-2 in cerebrospinal fluid (CSF). This indicates that the neurological symptoms were not caused by viral encephalitis but rather by CRS induced by COVID-19 infection (Perrin et al., 2021). The successful alleviation of CRS encephalopathy through the administration of steroids and intravenous immune globulin is a promising therapeutic strategy (Afshar et al., 2020; Pilotto et al., 2020).

In another clinical case, a man who was initially admitted with COVID-19 symptoms was later readmitted due to acute encephalopathy (Jang et al., 2020). Despite the absence of hypoxia, nasopharyngeal PCR confirmed SARS-CoV-2 infection. Upon readmission, the patient exhibited confusion, emesis, anorexia, and tremors, along with brain MRI findings suggestive of ischemia. Notably, CSF analysis did not detect SARS-CoV-2, ruling out encephalitis. The onset of encephalopathy correlated with increased levels of the inflammatory marker C-reactive protein, indicating a profound inflammatory reaction driven by innate immunity. This cytokine-associated toxicity, or cytokine storm, likely contributes to the development of encephalopathy through a cascade of proinflammatory cytokines crossing the BBB and triggering reactive gliosis, ultimately leading to brain scarring.

Despite elevated cytokine levels in COVID-19 patients, the virus interferes with the production of interferons, particularly type 1 interferon (IFN-I), which plays a crucial role in combating viral infections (Anjum et al., 2021). Although IFN-I is inhibited in lung tissue, its impact on brain tissue remains unclear. A case study involving a 39-year-old COVID-19 patient treated with IFN-β showed cognitive deficits despite recovery from respiratory failure, suggesting that the antiviral function of IFN-I may not extend to the brain (Umapathi et al., 2020).

Another study assessing CSF biomarkers in COVID-19 patients with neurological symptoms indicative of encephalopathy revealed elevated levels of neurofilament light chain (Nfl), indicating axonal injury (Edén et al., 2021). This suggests that neurological complications in COVID-19 patients may result from various mechanisms, including hypoxia-induced axonal injury.

In a study involving a sample of 175 individuals diagnosed with COVID-19, serum samples revealed increased levels of brain injury biomarkers, such as neurofilament light chain and glial fibrillary acidic protein (GFAP), in a manner that was dependent on the severity of the disease (Franke et al., 2021; Rachel et al., 2023). The observed elevation remained consistent even after a four-month period of observation, suggesting the presence of persistent brain injury. Increased levels of proinflammatory cytokines and autoantibodies against brain proteins, such as myelin-associated glycoproteins, were found to be correlated with these biomarkers (Rachel et al., 2023). Notably, a similar positive association between proinflammatory cytokines and biomarkers of brain injury was identified in a control group comprising 45 individuals diagnosed with influenza. This finding implies that the observed increases in these biomarkers may be associated with the severity of the infection rather than being specific to a particular virus (Rachel et al., 2023).

**Figure 1:**
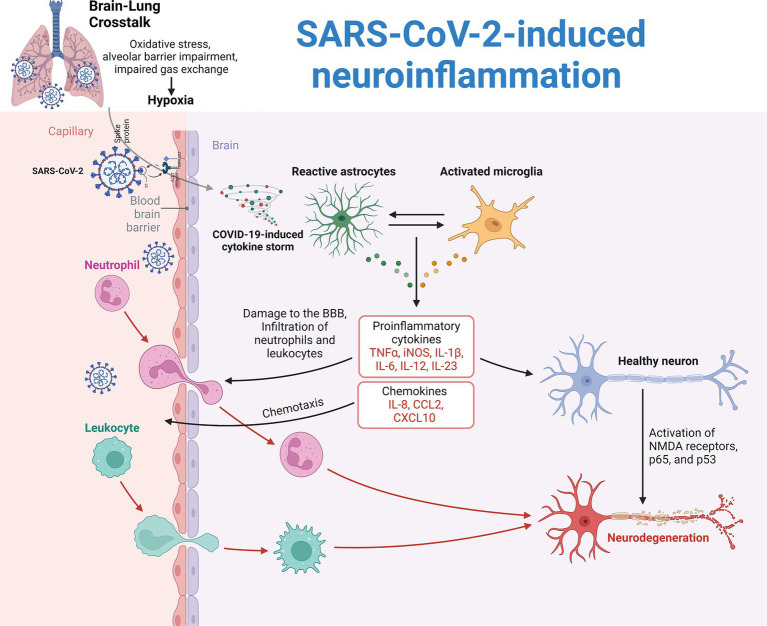
Pathogenesis of SARS-CoV-2-induced neuroinflammation. Two main theories explain the neuroinflammation observed in COVID-19 patients. The first theory is attributed to reduced systemic oxygenation, which leads to cerebral hypoxia and secondary brain damage, known as brain-lung crosstalk. The second theory involves the direct invasion of SARS-CoV-2, which triggers a hyperinflammatory response, causing a cytokine storm with inflammatory mediators such as IL-1β, IL-6, and TNF. This storm damages the blood–brain barrier (BBB), allowing immune cells and proinflammatory molecules to enter the brain. As a result, microglia and astrocytes become reactive. Microglia increase the production of reactive oxygen species, cytokines, and chemokines while reducing brain-derived neurotrophic factor (BDNF). Astrocytes increase glial fibrillary acidic protein (GFAP) and vimentin expression, leading to structural changes and reduced neurotransmitter recycling.

Certain patients with COVID-19 encephalopathy exhibit increased CSF concentrations of proinflammatory cytokines, such as interleukin 6, interleukin 8, and macrophage inflammatory protein-1 beta (MIP-1β). These findings may be associated with assessments of BBB integrity (Bartley et al., 2021; Rachel et al., 2023). Nevertheless, the occurrence of cytokines and disruption of the BBB extend beyond particular neurological phenotypes or abnormalities observed on brain imaging. Furthermore, there is a dearth of comparative studies involving control groups, such as COVID-19 patients who do not exhibit neurological symptoms (Douaud et al., 2022). There is limited evidence of SARS-CoV-2 in CSF (Abenza Abildúa et al., 2021; Rachel et al., 2023) (Figure 1).

In certain instances, there has been evidence supporting a humoral autoimmune mechanistic model (Rachel et al., 2023). In a limited number of cases, 23% of adolescents who were infected with SARS-CoV-2 and experienced neuropsychiatric complications displayed intrathecal antibodies against SARS-CoV-2 and neuronal autoantibodies on anatomical immunostaining. These individuals did not meet the criteria for encephalitis and had normal imaging and laboratory results (Starkey et al., 2017; Rachel et al., 2023). It is worth mentioning that a patient who exhibited a positive response to immunotherapy had antibodies specifically targeting transcription factor 4 that were isolated (Rachel et al., 2023). In three patients, severe myoclonus, the most prevalent movement disorder in patients with COVID-19 infection, and somnolence were observed in another case series. These patients had normal imaging and CSF levels (Helms et al., 2020; Rachel et al., 2023). An enhancement was noted subsequent to the administration of corticosteroids and/or plasma exchange, or both (Rachel et al., 2023).

Brain imaging associated with encephalopathy generally reveals normal findings, although it may sometimes display leptomeningeal enhancement, indicating inflammation. More severe cases, particularly in the intensive care setting, might show leukoencephalopathic alterations or microhemorrhages. These changes reflect damage to the white matter or small brain hemorrhages, often linked to the effects of severe COVID-19 or its treatments (Singh et al., 2022; Rachel et al., 2023). Another unusual finding, cytotoxic lesions in the splenium of the corpus callosum, has been reported in some cases, potentially resulting from cytokine-mediated glutamate release (Hosp et al., 2021; Huo et al., 2022; Rachel et al., 2023).

A meta-analysis conducted by Pyeong Hwa Kim et al. included 1,394 COVID-19 patients who underwent neuroimaging from 17 studies. Among these patients, 3.4% had COVID-19-related neuroimaging findings. The most common abnormality was related to the olfactory bulb and was observed in 23.1% of the patients. Among cerebral findings, white matter abnormalities were the most frequent (17.6%), followed by acute/subacute ischemic infarction (16.0%) and encephalopathy (13.0%). Critically ill patients had a significantly greater proportion of COVID-19-related neuroimaging findings than did other patients (Kim et al., 2021).

Functional imaging studies, such as positron emission tomography (PET) or functional magnetic resonance imaging (fMRI), can reveal abnormal patterns of brain metabolism or perfusion in COVID-19 patients with neurological symptoms. Frontoparietal or frontotemporal patterns are among the most commonly observed, suggesting that these regions might be more susceptible to the systemic effects of COVID-19 or related inflammatory responses (Liotta et al., 2020; Taquet et al., 2021; Rachel et al., 2023). Electroencephalography (EEG) in patients with COVID-19-related encephalopathy can reveal diffuse or focal slowing, which is indicative of brain dysfunction. In severe cases, patients might experience seizures or nonconvulsive status epilepticus, a critical condition requiring immediate intervention (Bartley et al., 2021; Rachel et al., 2023). CSF analysis is another valuable diagnostic tool. In COVID-19-related encephalopathy, CSF is often normal, suggesting a noninfectious cause. However, pleocytosis, an increase in white blood cells in the CSF, may indicate SARS-CoV-2-related or postinfective immune-mediated encephalitis. This condition requires further examination to exclude other possible infections caused by viruses or bacteria, as the treatment strategies can vary considerably (Xu et al., 2022; Rachel et al., 2023).

**Figure 2:**
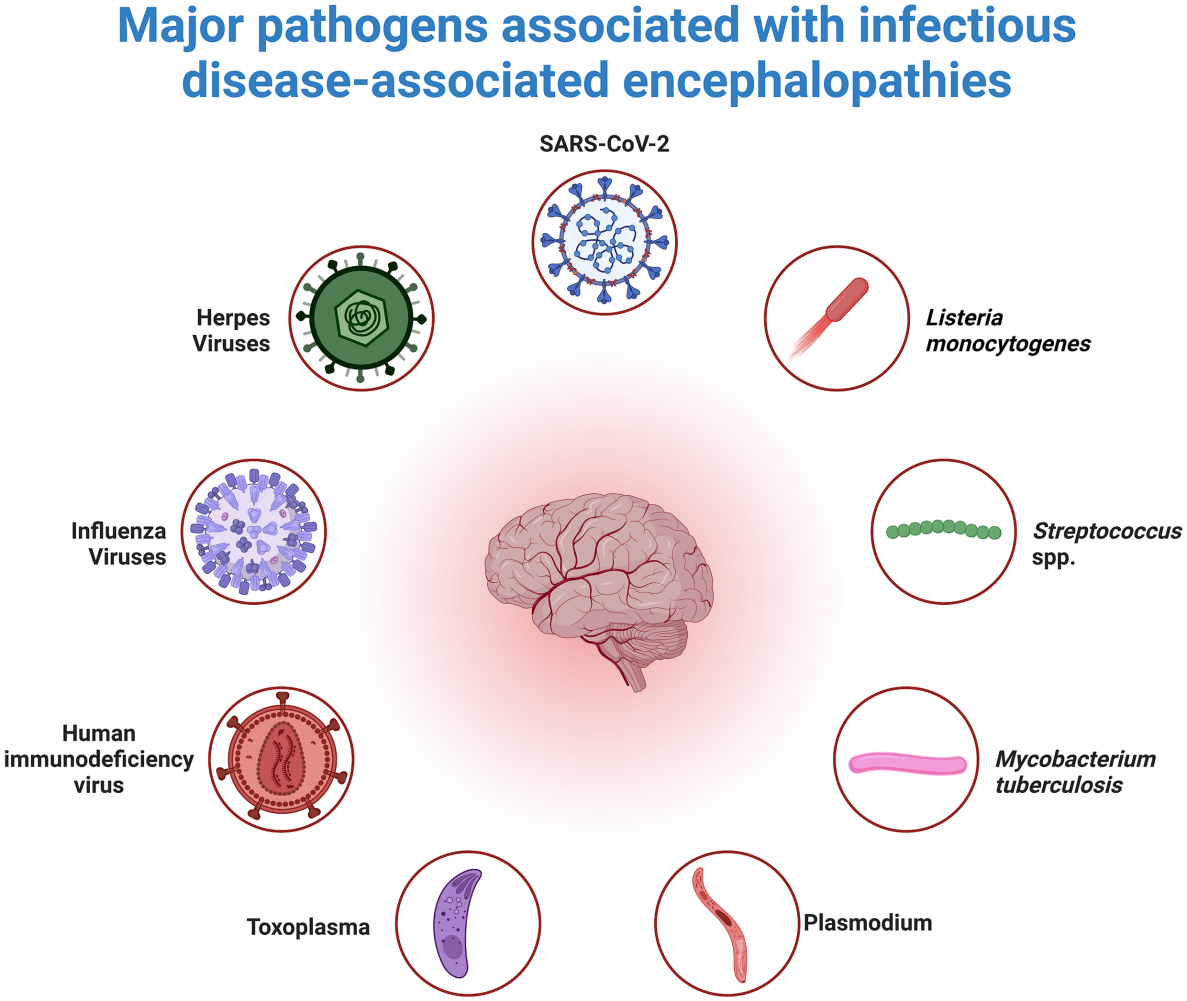
Overview of Pathogens that Cause Neuroinflammation. Neuroinflammation, a secondary inflammatory response in the brain, can occur as a result of peripheral infections caused by viruses, bacteria, or parasites. Inflammatory mediators that affect the brain endothelium and parenchyma as well as the subsequent response of brain cells to these mediators mediate this response.

Neuroinflammation, characterized by inflammatory processes within the central nervous system (CNS), is frequently observed in encephalopathies associated with infectious diseases. A diverse range of pathogens, including viruses such as influenza and herpes, protozoa such as Toxoplasma and Plasmodium, and bacteria such as *Mycobacterium tuberculosis* and *Listeria monocytogenes*, have been implicated in eliciting neuroinflammatory responses (Rock et al., 2008; Gilden and Nagel, 2016; Brasil et al., 2017; Tapajós et al., 2019). The pathogenesis of neuroinflammation involves the mediation of various molecules, including cytokines, chemokines, reactive oxygen species, and others (Barbosa-Silva et al., 2021). Cytokines have detrimental effects on brain function, particularly impacting the hippocampus (Milatovic et al., 2003; Richwine et al., 2008). Specifically, interleukin-1β (IL-1β) decreases synaptic strength and long-term potentiation in rodent hippocampi, influencing neuronal structure, synaptic adaptability, and memory and learning processes. In a study by Bellinger et al., interleukin-1β (IL-1β) was found to inhibit synaptic strength and reduce long-term potentiation in rodent hippocampi, thereby affecting neuronal structure, synaptic plasticity, and the processes underlying memory and learning (Lynch, 2002). Additionally, cytokines, notably IL-1β, hinder the signaling of brain-derived neurotrophic factor (BDNF), which is crucial for neuronal health (Tong et al., 2008). Furthermore, systemic administration of lipopolysaccharide (LPS) reduces the levels of BDNF, nerve growth factor (NGF), and neurotrophin-3, consequently affecting synaptic plasticity, memory, and neuronal survival (Guan and Fang, 2006) (Figure 2).

Glial reactivity also impacts neuronal cells, leading to a loss of support from glial cells. Astrocytes, for instance, regulate neurotransmitter levels such as gamma-aminobutyric acid (GABA), glutamate, and glycine in the synaptic space (Seifert et al., 2006). Astrogliosis, a consequence of glial reactivity, disrupts this regulation, resulting in glutamate toxicity (Heneka et al., 2015). Furthermore, glutamate toxicity can be exacerbated by the activation of indoleamine-2,3 dioxygenase (IDO), an enzyme expressed by microglia (Myint and Kim, 2014). Inflammatory mediators such as interferon (IFN)-γ and tumor necrosis factor (TNF)-α modulate IDO activity. Additionally, IDO plays a role in tryptophan-serotonin availability, suggesting that proinflammatory cytokines contribute to neurotransmitter imbalances (Schmidt et al., 2013).

## 3 Role of the gut-brain axis in patients with COVID-19

**Figure 3:**
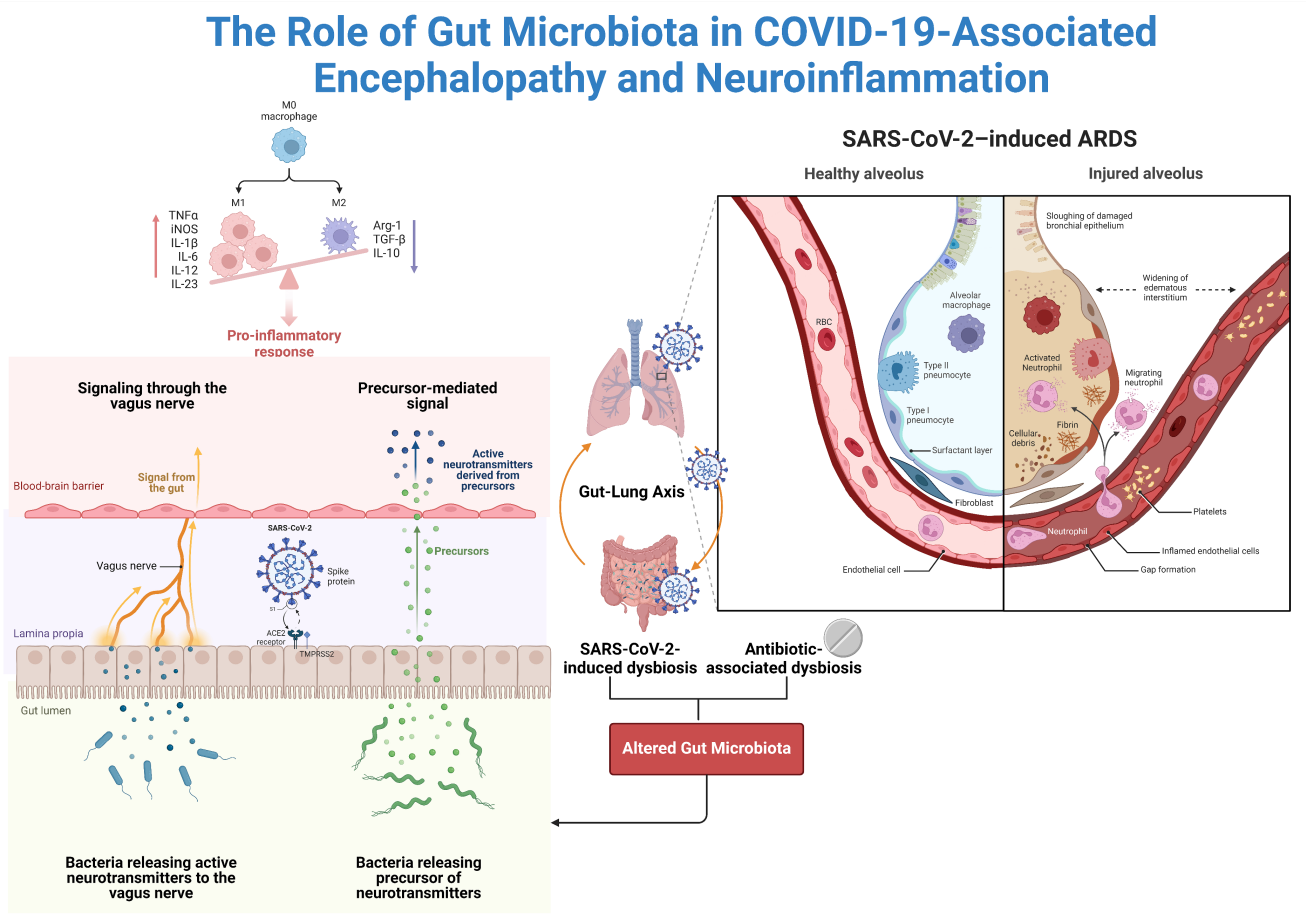
The Potential Impact of the Gut Microbiota on Neuroinflammation. Coronaviruses actively replicate in enterocytes since they highly express ACE2. The available data support a change in the microbiota in COVID-19 patients. Altered microbiota can cause intestinal barrier damage, systemic translocation of microbial products, and systemic inflammation. Additionally, the microbiota is connected to the brain via the vagus nerve and is referred to as the gut–brain axis.

Substantial evidence suggests that the SARS-CoV-2 virus can compromise the lining of the intestines and blood vessels within the brain through both direct and indirect mechanisms (Buzhdygan et al., 2020; Cardinale et al., 2020). Specifically, the virus relies on angiotensin-converting enzyme 2 (ACE2) receptors for cellular entry, which are abundant in the cells lining blood vessels in the brain and intestines (Hamming et al., 2004; Lu et al., 2020; Zhou et al., 2020). Studies have indicated that viruses can directly damage these cellular linings, induce inflammation, and trigger excessive immune responses known as cytokine storms, which can further exacerbate tissue damage (Elyaspour et al., 2021; Nicosia et al., 2021) (Figure 3).

The intestinal wall, which is rich in ACE2 receptors, is particularly susceptible to viral infiltration, leading to potential damage to the intestinal barrier and increased permeability (Cardinale et al., 2020). Additionally, COVID-19 frequently manifests with gastrointestinal disturbances, which can alter the balance of the gut microbiota toward a state that is proinflammatory and potentially harmful to neurological function (Kopel et al., 2020; Bernard-Raichon et al., 2022).

COVID-19 may cause substances with neurotoxic and neuroinflammatory properties to pass from the gut into the bloodstream due to a compromised intestinal barrier (Plummer et al., 2023). These substances may then enter the brain through weakened blood vessels, resulting in neuroinflammation caused by both direct viral damage and indirect effects of dysregulated immune responses (Plummer et al., 2023).

The previously established relationship between the gut microbiota and the CNS has been discussed as a potential explanation for the impact of SARS-CoV-2 on neurological functions (Monteleone et al., 2020). Reciprocal communication among the brain-gut-microbiota-immune axis is vital for maintaining immune equilibrium and the balance between Th17 and Treg cells (Luo et al., 2017; Pandiyan et al., 2019). Notably, the microbiota plays a significant role in this interplay and significantly influences these immune functions (Barnes and Powrie, 2009).

In the study by Viviani Mendes de Almeida et al., a notable increase in antibiotic-resistant Enterobacteriaceae strains was revealed in post-COVID-19 individuals compared to healthy controls. Additionally, reduced levels of short-chain fatty acids (SCFAs) are observed in fecal samples from post-COVID-19 patients, suggesting a potential link between altered gut microbiota and disease severity in COVID-19 patients (Mendes de Almeida et al., 2023).

SCFAs play a significant role not only in local effects within the colon and peripheral tissues but also in the intricate interplay between the gut and brain microbiota. The increased expression of monocarboxylate transporters (MCTs) in endothelial cells could facilitate the passage of SCFAs across the BBB, as evidenced by studies administering labeled SCFAs directly into the carotid artery of rats (Oldendorf, 1973; Vijay and Morris, 2014). Although research on the physiological levels of SCFAs in the brain is limited, all three SCFAs can be detected in human CSF, where they are present at higher concentrations than in peripheral blood (Li et al., 2017).

Furthermore, SCFAs appear to have a significant impact on the maintenance of BBB integrity. This is of utmost importance because it plays a critical role in regulating the transportation of nutrients and molecules into the brain, ultimately contributing to brain development and CNS homeostasis (Braniste et al., 2014). Research conducted on germ-free mice has shown that SCFAs control the production of tight junction proteins such as occludin and claudin, which in turn affect the permeability of the blood–brain barrier from the early stages of development to adulthood (Braniste et al., 2014). Nevertheless, the restoration of BBB integrity is achieved through the recolonization of these mice with complex microbiota or bacterial strains that produce SCFAs (Braniste et al., 2014).

Furthermore, previous studies conducted on cerebrovascular endothelial cells treated with propionate *in vitro* have indicated that SCFAs can alleviate the effects of LPS on vascular permeability (Hoyles et al., 2018). There is evidence indicating that SCFAs entering the CNS have neurological effects, although the specific mechanisms by which they work are still not fully understood. Animal studies have indicated a broad impact of SCFAs on various behavioral and neurological processes, potentially implicating them in significant stages of neurodegenerative and neurodevelopmental diseases (Silva et al., 2020).

Butyrate [also a short-chain fatty acid (SCFA)], an essential component in maintaining intestinal barrier integrity, plays a crucial role in this process (Candido et al., 1978). Furthermore, butyrate plays a crucial role in supporting the mucosal layer of the intestine. Additionally, it contributes to the process of histone acetylation, which alters the molecular composition of chromatin to facilitate transcription (Chriett et al., 2019). Moreover, butyrate has been shown to inhibit the expression of proinflammatory genes and the subsequent release of these genes induced by lipopolysaccharide (LPS) in endothelial cells. It has been proposed that the reduction in bacteria that produce butyrate may exacerbate the damage to gut epithelial cells caused by SARS-CoV-2, resulting from insufficient downregulation of these inflammatory mechanisms (Li et al., 2021).

Additionally, research has shown that butyrate can improve the body’s natural immune response to viral infections by activating the Toll-like receptor signaling pathway. This results in increased levels of interleukin-1β, interferon regulatory factor-7, and interferon-alpha/beta receptor expression at both the mRNA and protein levels. Therefore, butyrate likely plays a role in the innate immune response to SARS-CoV-2 (Li et al., 2021).

LPS, an important constituent of the outer membrane of gram-negative bacteria in the gastrointestinal tract, induces systemic inflammation and stimulates microglia in the brain (Raetz and Whitfield, 2002; Brown, 2019). While elevated levels of this endotoxin have been detected in COVID-19 patients admitted to hospitals, its precise contribution to neuroinflammation in these patients is still unclear (Teixeira et al., 2021). Research conducted on rats has shown that LPS can directly enter the brain and bind to receptors on endothelial cells at the interface between the blood and brain. This process exacerbates damage to the endothelium caused by viruses and cytokines (Vargas-Caraveo et al., 2017). Importantly, LPS interferes with the BBB in specific areas of the brain, impacting regions that are essential for complex cognitive processes such as the thalamus and frontal cortex (Jung and Haier, 2007; Vakhtin et al., 2014).

**Figure 4:**
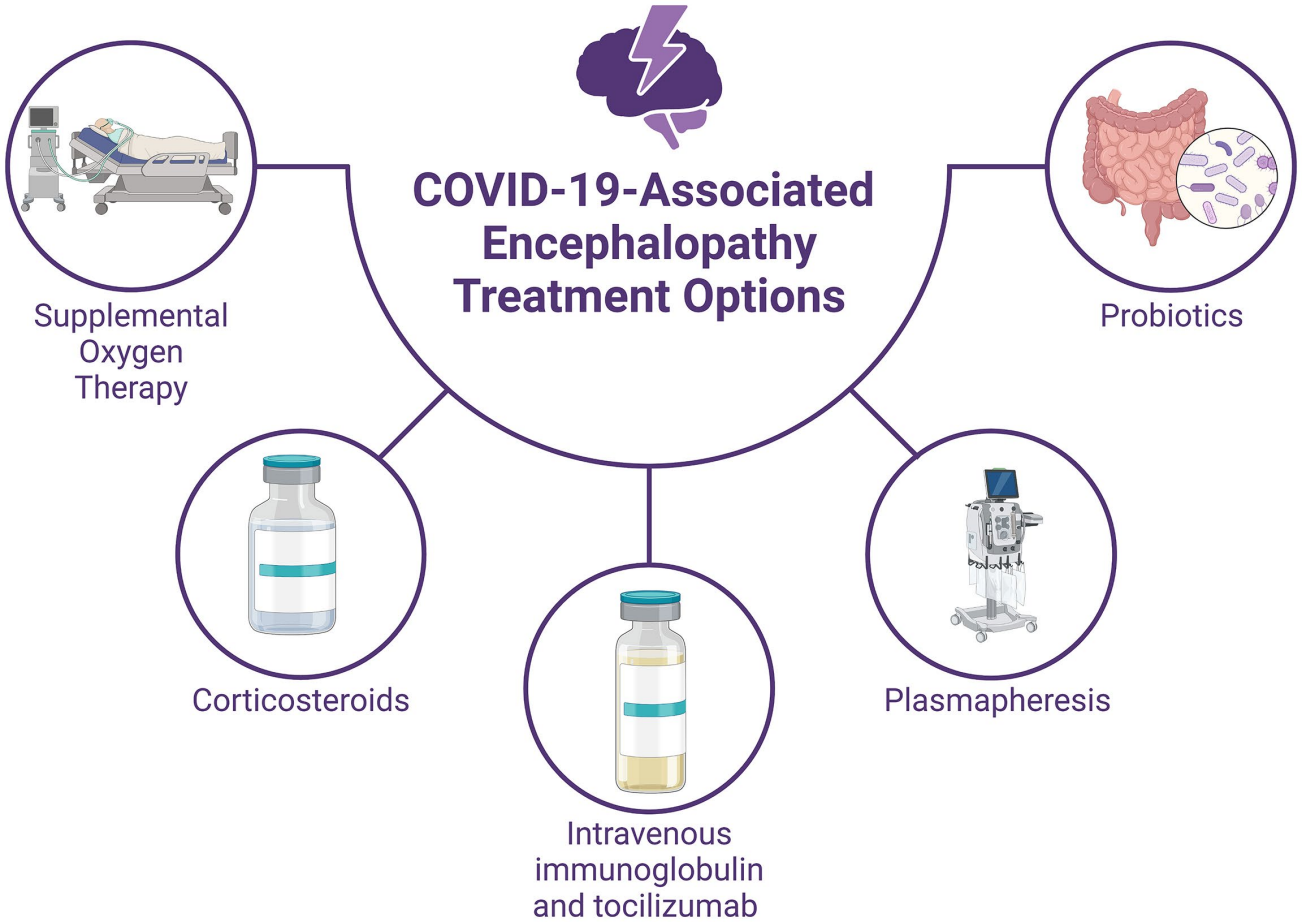
Treatment Options for COVID-19-associated Encephalopathy. Supportive therapy includes supplemental oxygen to address hypoxemia and immune modulation with high-dose corticosteroids and intravenous immunoglobulin to manage the systemic inflammatory response. Plasmapheresis can help reduce proinflammatory marker levels and improve consciousness in severe cases. Immunomodulation involves tocilizumab, which may reduce the need for mechanical ventilation in some patients but does not guarantee improved survival. Probiotic therapy with strains such as *Lactobacillus plantarum* and *Pediococcus acidilactici* has shown potential in reducing viral load and improving symptoms. Studies suggest that probiotic-supplemented patients may have shorter hospital stays and better outcomes.

Once overshadowed by LPS, peptidoglycan (PGN) has emerged as a gram-positive counterpart contributing to systemic endotoxicity (Myhre et al., 2006). This shift is attributed to the increasing incidence of sepsis caused by gram-positive organisms (Mayr et al., 2014). PGN concentrations in blood plasma reflect intestinal permeability due to various conditions, such as ischemia and hemorrhagic shock (Tsunooka et al., 2004). Gut-derived PGN has been linked to inflammatory and autoimmune conditions, including multiple sclerosis, and has shown neurotoxic effects in animal models, triggering microglial and astrocytic nitric oxide production and leading to neuronal cell death (Boje and Arora, 1992; Buskila et al., 2005). Beyond its role in innate immunity, PGN has implications for neurodevelopment and associated disorders such as autism spectrum disorder (Arentsen et al., 2017; Gonzalez-Santana and Diaz, 2020). Elevated PGN concentrations have also been noted in hospitalized COVID-19 patients, potentially contributing to persistent neuroinflammation and neurotoxicity along the gut-brain axis (Plummer et al., 2023) (Figure 4).

## 4 Current and potential therapeutic options

The standard approach for managing encephalopathy in COVID-19 patients primarily involves supportive measures such as supplemental oxygen therapy and immune modulation (Wiersinga et al., 2020). Immune modulation therapy, including the administration of high-dose corticosteroids (intravenous methylprednisolone at a dosage of 500 mg to 1 g per day for 5 days) and intravenous immunoglobulin (at a dosage of 0.1–0.5 g per kg per day for 5–15 days), is prioritized over antiviral treatment due to the systemic inflammatory response induced by SARS-CoV-2 (Galeotti et al., 2020; Pilotto et al., 2020; Pugin et al., 2020). Additionally, repeated plasmapheresis has demonstrated efficacy in enhancing consciousness and reducing the levels of proinflammatory markers in the bloodstream (Dogan et al., 2020). Critically ill patients with encephalopathy are often managed in the intensive care unit (ICU), where mechanical ventilation may be necessary. Anti-epileptic drugs should be initiated both as abortive and prophylactic therapy in these patients if they exhibit altered mental status, seizures, or subtle muscular spasms (Shah et al., 2021). However, close monitoring for adverse effects and potential drug interactions is crucial, given the significant respiratory and cardiac risks associated with antiepileptic medications (Asadi-Pooya, 2020). The increased tendency for blood clot formation observed in COVID-19 patients can be effectively managed through the administration of anticoagulant drugs such as heparin. This treatment approach has been linked to improved outcomes, particularly in individuals with significantly elevated levels of a marker called D-dimer (Horie et al., 2020). Additionally, in COVID-19 patients not requiring mechanical ventilation, tocilizumab has been identified as a medication capable of lowering the risk of progression to the need for mechanical ventilation or death. However, it should be noted that tocilizumab does not confer a survival benefit in these patients (Salama et al., 2021).

Another adjuvant therapeutic option can be probiotics, which are defined as beneficial microorganisms capable of surviving and proliferating within the gastrointestinal tract (Lehtoranta et al., 2014). They exert protective effects against a spectrum of pathogens while exhibiting varied benefits based on their specific strains. Through their interaction with the host immune system, probiotics elicit a response involving the production of both anti-and proinflammatory cytokines such as IL-10, IL-12, IL-17, and interferon-α (IFN-α) upon encountering infectious agents (Azad et al., 2018; Kalam and Balasubramaniam, 2024). This immunomodulatory action encompasses two pivotal mechanisms: an immunostimulatory effect activating IL-12 and promoting the activity of natural killer (NK) cells, T1 helper cells, and T2 helper cells (Th1 and Th2) against harmful pathogens and an immunoregulatory effect fostering the production of IL-10 and regulatory T cells (Tregs) by stimulating various immune cells, including Th-2 cells, B cells, dendritic cells (DCs), and monocytes, thereby orchestrating an adaptive immune response within the human host (Chiba et al., 2010; Kalam and Balasubramaniam, 2024).

A clinical study was conducted to evaluate the effectiveness of a combination of probiotics in outpatients who tested positive for COVID-19 (Kalam and Balasubramaniam, 2024). The mixture consisted of strains of *Lactobacillus plantarum* and *Pediococcus acidilactici* in equal proportions. All participants exhibited favorable tolerance to the probiotics during the trial. It is worth mentioning that the treatment group demonstrated a significant decrease in viral load in the nasopharyngeal region, as well as a reduction in both gastrointestinal (GI) and non-GI symptoms, compared to the placebo group (Kalam and Balasubramaniam, 2024). Interestingly, the individuals who received treatment exhibited elevated levels of antibodies specific to SARS-CoV-2 compared to those in the control group. However, no significant changes were observed in the composition of the fecal microbiota in either group. The effectiveness of probiotics may primarily depend on their interaction with the immune system of the host rather than the overall composition of the microbiota (Chiba et al., 2010; Kalam and Balasubramaniam, 2024).

Additionally, a study was conducted to evaluate the effectiveness of various strains of beneficial bacteria, including *Lactobacillus*, *Bifidobacterium*, and *Enterococcus*, in COVID-19 patients across a spectrum of disease severities. Patients received a dose of 1.0 × 10^7^ CFU of probiotics alongside standard care (Kalam and Balasubramaniam, 2024). The results revealed that patients supplemented with probiotics exhibited improved clinical outcomes and a shortened duration of hospitalization compared to those receiving standard care alone (Zhang et al., 2021; Kalam and Balasubramaniam, 2024).

## 5 Conclusion

Encephalopathy associated with COVID-19 is characterized by neuroinflammation, which can manifest even without direct viral invasion into the central nervous system. Neuroinflammation, which is mediated by cytokines, chemokines, and reactive oxygen species, plays a central role in the pathogenesis of encephalopathy. COVID-19 induces encephalopathy primarily through two main mechanisms: hypoxia and the overproduction of cytokines.

SARS-CoV-2 also affects the intestinal epithelium, leading to gut dysbiosis, which can exacerbate neuroinflammation through the gut-brain axis. This altered balance in the gut microbiota may allow neuroinflammatory substances to enter the bloodstream and reach the brain.

Probiotics show potential for mitigating symptoms associated with COVID-19. Studies have shown improvements in immune modulation, reduced viral load, and improved clinical outcomes in COVID-19 patients who receive probiotics. However, further research is necessary to understand the mechanisms involved and optimize the use of probiotics in managing COVID-19-associated encephalopathy.

## Author contributions

KD: Writing – original draft, Conceptualization. PP: Writing – original draft, Visualization. OK: Supervision, Writing – review & editing.
